# ENERGY expenditure of COmmuting to school (ENERGYCO): protocol for a cluster randomized controlled trial

**DOI:** 10.3389/fpubh.2025.1467227

**Published:** 2025-03-14

**Authors:** Pablo Campos-Garzón, Víctor Manuel Valle-Muñoz, José Manuel Segura-Díaz, Manuel Ávila-García, Romina Gisele Saucedo-Araujo, Ana Ruiz-Alarcón, Francisco David López-Centeno, Unai A. Pérez De Arrilucea Le Floc’h, Juan M. A. Alcantara, Luis Miguel Medel-Carbonell, David Rodriguez-Sanchez, Ana Ramírez-Osuna, Marina Castillo-Barragán, Estela Águila-Lara, Francisco Javier Huertas-Delgado, Manuel Herrador-Colmenero, Sandra Mandic, Palma Chillón, Yaira Barranco-Ruiz, Emilio Villa-González

**Affiliations:** ^1^Faculty of Health Sciences, University of Lethbridge, Lethbridge, Alberta, Canada; ^2^Department of Physical Education and Sports, Faculty of Sport Sciences, Sport and Health University Research Institute (iMUDS), University of Granada, Granada, Spain; ^3^Department of Didactics of Musical, Plastic and Corporal Expression, University of Jaén, Jaén, Spain; ^4^Inmaculada Teacher Training Centre, Sport and Health University Research Institute (IMUDS), University of Granada, Granada, Spain; ^5^Faculty of Sport Sciences, University Isabel I, Burgos, Spain; ^6^Department of Specific Didactics, Faculty of Education, University of La Laguna, San Cristóbal de La Laguna, Santa Cruz de Tenerife, Spain; ^7^Sport and Health University Research Institute (iMUDS), University of Granada, Granada, Spain; ^8^Universidad Europea de Valencia, Faculty of Health Sciences, Nursing Department, Research Group Quality of Life and Health, Valencia, Spain; ^9^Department of Health Sciences, Institute for Innovation & Sustainable Food Chain Development, Public University of Navarre, Pamplona, Spain; ^10^Navarra Institute for Health Research, IdiSNA, Pamplona, Spain; ^11^Centro de Investigación Biomédica en Red Fisiopatología de la Obesidad y Nutrición (CIBERobn), Instituto de Salud Carlos III, Madrid, Spain; ^12^School of Sport and Recreation, Faculty of Health and Environmental Sciences, Auckland University of Technology, Auckland, New Zealand; ^13^AGILE Research Ltd., Wellington, New Zealand

**Keywords:** youth, active transport, cycling, resting metabolic rate, energy expenditure

## Abstract

**Introduction:**

This article outlines the rationale and methodology of the ENERGY expenditure of COmmuting to school study (the ENERGYCO study), a cluster-randomized controlled trial. The ENERGYCO study is divided into two phases: Phase I will aim; to assess the physical activity energy expenditure (PAEE) of different modes of commuting to school (i.e., walking, cycling, and motorized-vehicle) using indirect calorimetry in Spanish adolescents; and Phase II will aim to assess the effect of a school-based cycling intervention on resting metabolic rate and PAEE, as well as on other physiological, physical, and psychosocial outcomes on Spanish adolescents.

**Method:**

For Phase I, a convenience sample of ~50 adolescents will be recruited. These participants will have their PAEE assessed in three different modes of commuting. Regarding phase II, a total of 300 adolescents from different schools in three Spanish cities will participate in this cluster randomized controlled trial. As many schools as necessary to meet the target sample will be included. In addition, each school will be randomized as either an intervention or control group. Participants from intervention schools will be asked to complete a school-based cycling intervention, while participants from control schools will be asked to continue their same habits for 8 weeks. The school-based cycling intervention will last for 8 weeks and will include *Bikeability* sessions, along with encouragement strategies to increase adherence to cycling to and from school.

**Conclusion:**

The ENERGYCO study will provide novel insights into the PAEE associated with different modes of commuting to school using indirect calorimetry, as well as a comprehensive overview of how an 8-week school-based cycling intervention impacts resting energy expenditure, daily energy expenditure, and the physical and psychosocial health of adolescents.

## 1 Introduction

Resting metabolic rate (RMR) constitutes the primary component of 24-h energy expenditure, representing the energy requirement for sustaining bodily functions while at rest in thermoneutral conditions (1). Given, fat-free mass (FFM) serves as a robust predictor of RMR in adolescent population (2, 3), exercise-induced weight loss is an important research topic since it leads to increases RMR by increasing the fat free mass component (4). However, studies in the adolescent population to modify the RMR have focused mainly on resistance or aerobic exercise (5, 6), without taking into account how regular physical activity (PA) might affect the RMR. Understanding PA energy expenditure (hereinafter referred to as PAEE) as the result of any muscular movement due to PA (7), the interaction of intensity, duration, and frequency of PA determines the total PAEE. In that sense, the American College of Sports Medicine states that exercise and health-oriented PA should be performed consistently to obtain its associated benefits. Nevertheless, nowadays there is an energy imbalance in the youth population, with high energy intake and low PAEE (8). This fact may have led to an increase in obesity in the young population worldwide, by 5.6% in girls and 7.8% in boys between 1975 and 2016 (9). In fact, the World Health Organization (WHO) has considered the overweight and obesity as a global public health concern (10). Although overweight and obesity have a multifactorial origin (9, 11), the literature recommends increasing PAEE through regular PA, as it is the most easily modifiable component of 24-h energy expenditure (11), to address the current situation. Therefore, interventions targeting young populations should aim to increase PAEE to restore or maintain energy balance (12).

In this regard, given that the young population commutes to and from school at least twice on a school day, promoting active modes of commuting, mainly walking or cycling (13), has been considered as an effective strategy to improve their health (14). Indeed, scientific evidence shows that active commuting to and from school (ACS) is associated with higher levels of total PA (15), during weekdays (16), and it even appears that those adolescents who walk to school also are more active in other contexts (17, 18). In fact, such is its impact on PA levels, that in the meta-analysis conducted by Campos-Garzón et al. (19), they indicated that actively commuting to and from school may help to complete 48% of the PA recommendations in young population. Nevertheless, few studies have analyzed the PAEE related to ACS (12, 20) and to the best of our knowledge, no study has analyzed how ACS impact on RMR. Moreover, while it is well known that walking and cycling are active modes of commuting to school, they can provide different health benefits (14). Cycling to school could be more related to physical fitness and physiological improvements such as, increasing in cardiorespiratory fitness levels and overall health (14), contributing to reducing the risk of cardiovascular diseases, obesity, diabetes or depression (14, 21–24). Therefore, although ACS has several associative benefits, it is important to know the differences between walking and cycling when promoting it (25).

Despite the limited number of studies analyzing PAEE related to ACS, a significant gap remains in accurately assessing PAEE during free-living behaviors. Indirect calorimetry is widely recognized as the gold standard for measuring PAEE, as it directly quantifies oxygen consumption and carbon dioxide production, providing continuous, real-time data that capture the complex metabolic responses during activity (26). This direct measurement offers a significant advantage over methods that rely on predictive equations or self-reported data, which often oversimplify the dynamic and context-specific nature of free-living behaviors (26). Previous studies have frequently relied on stationary or semi-portable indirect calorimetry systems that are not well-suited for field applications (27). Although portable metabolic systems have been developed to facilitate outdoor assessments, their high cost and operational complexity limit their widespread use in educational and field contexts (27). As an alternative, researchers have employed established equations, such as those proposed by Riebe et al. (28) and Pandolf et al. (29). However, these equations were derived under highly controlled laboratory conditions (e.g., typically on treadmills) and, consequently, do not account for the variability of environmental factors (e.g., changes in speed, terrain, and weather), which critically influence PAEE in real-life settings, particularly in ACS (30). Another common practice for analyzing PAEE related to ACS has been through estimates based on counts measured by accelerometers (20, 31). However, accelerometers may not be the most appropriate devices for measuring PAEE in the context of ACS (32). Moreover, a recent study compared the PAEE associated with walking to that of using an e-scooter (33). However, this study was conducted under laboratory conditions, making it impossible to account for environmental factors that could influence PAEE. It is well known that the availability of safe and accessible routes directly correlates with increased PA among adolescents, which could, in turn, lead to higher PAEE (34). Additionally, PA levels may depend on adolescents’ perceptions in their commuting environment, considering factors such as fear of accidents, infrastructure, and social norms related to active commuting behaviors (35). Therefore, it is crucial to compare the PAEE associated with different modes of commuting to school in free-living conditions. Furthermore, developing a context-specific equation that incorporates key commuting characteristics (e.g., speed and distance) would provide a more accurate estimation of PAEE associated with this behavior.

Furthermore, ACS interventions have primarily been conducted in children (i.e., from 6 to 11 years) rather than in adolescents (i.e., from 12 to 17 years) as well as promoting walking rather than cycling. These interventions have primarily focused on changes in the mode of commuting, frequency of commuting, or increased PA levels (36–39) rather than assessing the effects on 24-h energy expenditure components (i.e., RMR and PAEE). In addition, interventions to improve RMR or PAEE have mainly consisted of resistance or endurance trainings (5, 11), being these activities performed out of school. To the best of our knowledge the effect of a school-based cycling intervention on the RMR and daily PAEE of adolescents has not been studied to date. In fact, the assessment of the effects of a school-based cycling intervention could be particularly important to consider as a suitable strategy to increase the RMR and re-balance PAEE in the adolescent population. In addition, a school-based cycling intervention will not only produce effects at physiological level (i.e., RMR or PAEE), but it will also allow to deepen the effects of this mode of commuting to school at physical or psychosocial level in the adolescent population. It has been shown that cycling to school is associated with greater grip strength and vertical jump (40), but the association does not imply causality. Similar results occur at the psychosocial level, where cycling to school has been associated with greater well-being or increased social relations compared to use passive passively commuting to school in adolescents (41). Moreover, there is controversy as to whether cycling to school could improve young people’s body composition (14). Thus, a school-based cycling intervention is needed to increase and improve the evidence of the effects of cycling to school in physiological, physical, and psychosocial outcomes.

Therefore, this protocol describes the rationale and methodology of the ENERGY expenditure of school Commuting study (The ENERGYCO study). ENERGYCO project will address the gaps and limitations in the existing scientific literature regarding the PAEE that occurs during cycling to school. Considering the previous literature and existing gaps, the ENERGYCO project will be divided into two phases. Phase I will focus on using a portable metabolic system to assess PAEE associated with different modes of commuting (i.e., walking, cycling, motorized vehicles) under free-living conditions in Spanish adolescents. Additionally, Phase I will serve to develop a predictive equation that will be used in Phase II to measure PAEE associated with different modes of commuting to and from school. Additionally, considering the ongoing debate regarding the health benefits of ACS and the scarcity of studies examining the effects of school-based cycling interventions on RMR and PAEE, the Phase II will aim to assess the effects of a school-based cycling intervention on RMR and PAEE, as well as on other physiological (i.e., basal heart rate, blood pressure, and body temperature), physical (i.e., PA levels, anthropometric measures, body composition, physical fitness, and motor competence), and psychosocial outcomes (e.g., perceived barriers to active commute to school, self-esteem, and mental health) on Spanish adolescents. It is hypothesized that, in Phase I, using a portable metabolic system under free-living conditions will provide accurate and distinct measurements of PAEE associated with different commuting modes in Spanish adolescents. Specifically, we hypothesize that these measurements will highlight significant differences in PAEE across commuting modes, offering a more precise understanding of how commuting impacts adolescents’ physical activity levels. Additionally, we expect the data collected in Phase I to contribute to the development of a valid and reliable predictive equation for estimating PAEE during commuting to and from school. we hypothesize that a school-based cycling intervention will lead to a significant increase in both RMR and PAEE compared to a control group. We further hypothesize that the intervention will improve physiological outcomes (e.g., reductions in basal heart rate), physical outcomes (e.g., enhanced physical fitness and motor competence), and psychosocial outcomes (e.g., increased self-esteem) among Spanish adolescents.

## 2 Methods

### 2.1 Study design and participants

The ENERGYCO study is a cluster randomized controlled trial to be conducted in high schools from three Spanish cities (Granada, Jaén, and Almería), where the schools will be the units of randomization, and individuals within the schools will be the units of analysis (participants). This project will consist of two main phases: Phase I will present a cross-sectional design which will take place before starting the Phase II. A cross-sectional approach will be adopted due to its suitability for capturing a snapshot of key variables of interest at a specific point in time, aligning with the objectives of this phase (i.e., comparing PAEE associated with different modes of commuting to school and developing predictive equations for measuring PAEE in Phase II). In contrast, for Phase II a cluster parallel-arm design will be employed to allocate interventions effectively to larger groups while accounting for potential intra-cluster variability, enhancing the external validity and generalizability of the results. The ENERGYCO study has been approved by the Review Committee for Research Involving Human Subjects at the University of Granada (Reference: 2496/CEIH/2021) and registered with a ClinicalTrials.gov ID: NCT06414668. In addition, the present study protocol has been written in accordance with the Standard Protocol Items: Recommendations for Interventional Trials (SPIRIT) statement (See Supplementary material 1).

### 2.2 Study population

**Figure 1:**
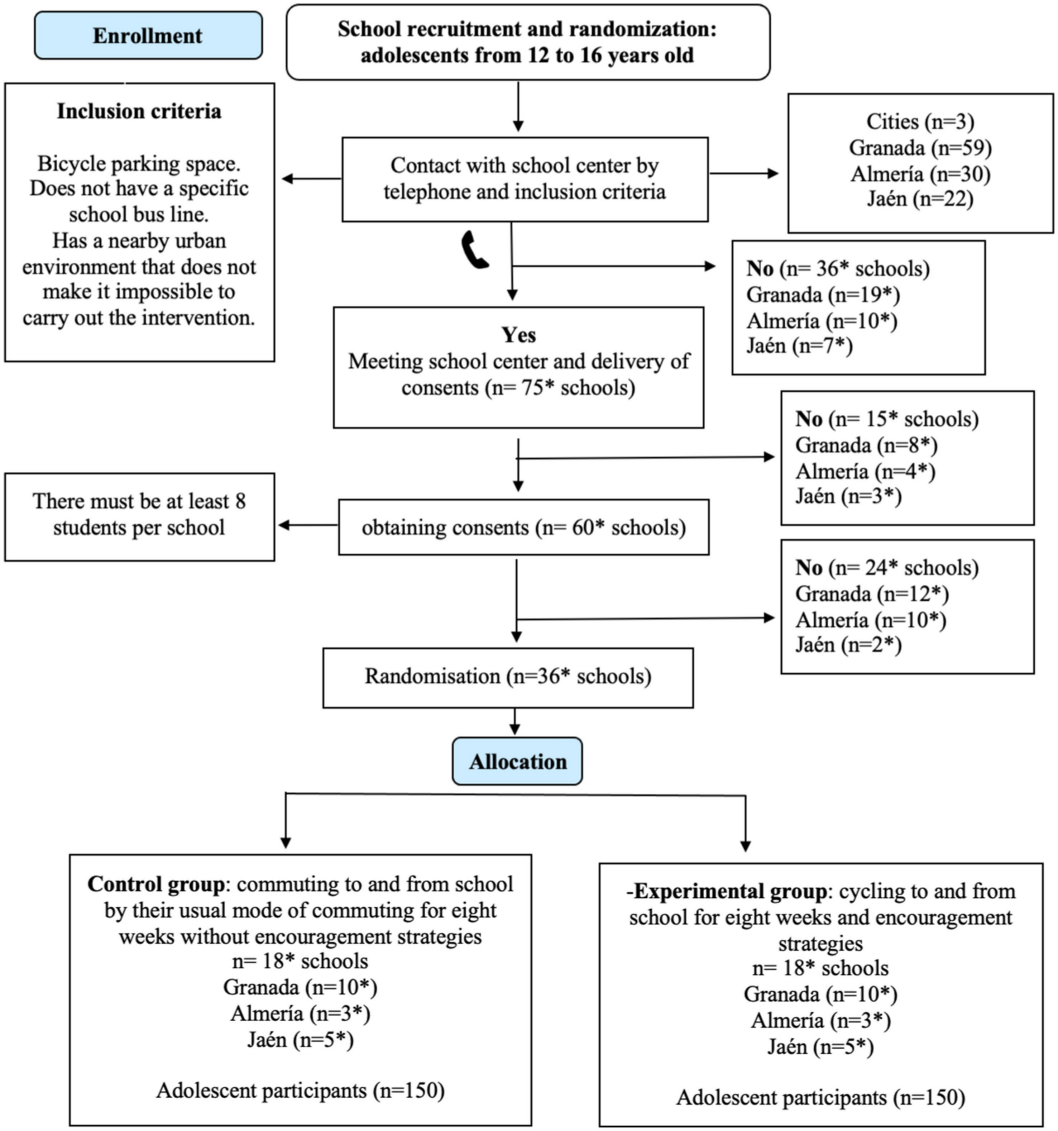
School recruitment plan flow chart of the ENERGYCO study. *Expected number during the school recruitment process.

In the Phase I of the ENERGYCO study, a convenience sample of 50 students (12–16 years old) will be recruited to assess the PAEE of different modes of commuting to school (i.e., walking, cycling, and motorized-vehicle). For Phase II, the ENERGYCO study will recruit a random sample of students (*n* = 300) from the three Spanish cities (i.e., Granada, Almería, and Jaén). The intervention aims to assess the effects of a school-based cycling intervention in the RMR and PAEE related to cycling to school, as well as its impact on others physiological, physical, emotional, and psychosocial outcomes in Spanish adolescents. The randomization process is presented in Figure 1.

### 2.3 Trial management groups

The trial management group will be composed of a steering committee of investigators responsible for overseeing all aspects of trial implementation. Their primary responsibilities include coordinating participant recruitment, ensuring compliance with study protocols, and monitoring data quality. Moreover, given the wide range of variables included in the ENERGYCO project, each investigator will be assigned a specific area of coordination: physiological variables (e.g., RMR and PAEE), physical variables (e.g., sedentary time and PA levels), psychosocial variables (e.g., self-esteem, and mental health), among others. Although the inclusion of additional oversight committees was considered, the manageable scope of this trial, combined with the expertise of the current steering committee, is considered sufficient to ensure proper trial governance and robust data quality control.

### 2.4 Procedures

#### 2.4.1 School recruitment and randomization

In Phase I, a convenience sample of approximately 50 students will be recruited. A call to action will be used to engage adolescents through the development of infographics and information about the aims of this phase, which will be disseminated through social media, personal contacts, or emails. Since these tests will be conducted outside the school timetable, no schools will be directly contacted. Instead, parents or legal guardians will be responsible for contacting the research team if their children are interested in participating in the study and they provide their consent. In this call to action, incentives, such as activity wristbands will be given away to participating adolescents. For Phase II, a specific selection protocol will be implemented through a random draw. A list of all public secondary schools in each of the participating cities of the government educational institutions in Andalucía will be obtained. The schools in the three cities (Granada, Almería, Jaén) will be contacted by phone, and if they are interested in participating, they will have to meet a series of inclusion and exclusion criteria presented in Table 1. During the call, a meeting will be arranged with interested schools to explain to the management team and teaching staff the main objective of the study, as well as to obtain official consent to participate in the project from the school. After that, the research staff with the help of Physical Education teachers will recruit participants and their families, by visiting selected classes and explaining the study to prospective adolescent participants and finally requesting the family’s consent. The inclusion and exclusion criteria for the participants are presented in Table 2. To complete the sample of students in the intervention group, several schools may be needed, as not all students will be able to participate in the school-based cycling intervention due to family reasons, distance, predisposition, etc. To encourage as many students as possible from the intervention school to participate (with their families’ consent) in the school-based cycling intervention, an incentive system will be planned similar to other studies (42, 43). This incentive will consist of a voucher to be redeemed for sports equipment, provided that the participant has completed the 8 weeks of intervention and presents valid data at both baseline and follow-up. Once finished the baseline assessment, the participating schools will be randomly assigned through a draw to either a control or experimental group.

**Table 1:** Schools’ inclusion and exclusion criteria.

Inclusion criteria	Exclusion criteria
The school has a specific space for parking bicycles and allows their parking	The school does not have specific infrastructure to park or store bicycles
The school has at least two classes per grade	The school offers bus transportation to the adolescents
At least eight students from the school will agree to participate in the study	The school is already participating in other interventions focused on promoting healthy behaviors
Schools from urban areas	The school has implemented an intervention to encourage commuting to school within the last year
	Schools from rural areas

**Table 2:** Participants’ inclusion and exclusion criteria.

Inclusion criteria	Exclusion criteria
Adolescents’ females and males aged from 12 to 17 years old (both ages included)	Adolescents younger than 12 years or older than 17 years
Healthy participants who can ride a bicycle safely and without difficulty.	Participants with any injury or medical condition that prevents safe participation in cycling to and from school
Be able to cycle to and from school at least 3 days per week during eight consecutive weeks (Intervention group)	Insufficient ability to ride a bicycle or inability to cycle to and from school at least 3 days a week (Intervention group)
Not to cycle to and from school more than 3 days a week during the last year	Participants who have cycled to and from school more than 3 days a week during the past year
To present a valid signed consent from their parents or legal guardians	Participants without a valid, signed parental or guardian consent form
To present valid data at baseline and follow-up from all the outcomes assessed	Participants who do not provide valid data for all assessed outcomes at baseline and follow-up

#### 2.4.2 Sample size calculations

For Phase II, at least 250 participants are needed to ensure an acceptable sample size, assuming 5% precision (*α* err prob) and a very high statistical power of 95%. (Error 1-*β* err prob) (44). This calculation has been made considering a study objective where the effect of the intervention program on the different health variables of young people is evaluated (e.g., RMR, EE, PA levels, body composition, muscle strength). In this study there will be two main comparison groups (experimental group and control group) and for this the ANCOVA statistical analysis will be performed: fixed effects, main effects, and interactions (F test) and with an effect size = 0.25. It is assumed that there will be dropouts during the school-based cycling intervention (≈15% in the experimental group), so ≈300 adolescents will be recruited from different schools in the three Spanish cities. Following the forecasts in Figure 1, 100 adolescents would be needed in each city. The groups randomization will be done at a 1:1 ratio at city level (i.e., from each city, 50 participants will be randomized to intervention and 50 to control groups), as other studies have done before (45). Nevertheless, due to the low prevalence of cycling to school in Spain (46), we will try to recruit as many schools as necessary in each city to reach the number of 100 adolescents.

### 2.5 Description and rationale of the ENERGYCO study

In Phase I the PAEE of different modes of commuting will be quantified by analyzing each participant individually in different controlled environments. For phase II, after recruiting schools and participants, an eight-week school-based cycling intervention will be implemented. The choice of an eight-week duration was based on the results reported in the systematic review by Dorothea et al. (47) on the effects of cycling interventions in a school setting. For example, although interventions lasting longer than 8 weeks demonstrate positive changes in physical fitness (43, 48), they also exhibit lower adherence, with a higher dropout rate among participants post-intervention. Therefore, considering that interventions lasting 4 to 8 weeks show effects on self-efficacy (49) or PA levels (50) and report a lower dropout rate, an eight-week duration was chosen for the school-based cycling intervention. The intervention will consist of adolescents having to cycle to and school for eight consecutive weeks. To encourage participants to complete the intervention, they will take part in a *Bikeability* training and different encouragement strategies will also be used during this period. Moreover, the ENERGYCO school-based cycling intervention shows relevant factors described in the current National Education Law for the curriculum of Compulsory Secondary Education. This approach is based on strategies proposed by the Active Living by Design Community Action Model (51), which involves preparing through the development of interventions, promoting education, and motivating students to cycle anywhere, as well as organizing activities with incentives and programs to engage participants. Regarding the control groups, participants in these schools will be asked to continue their daily habits. In addition, at the end of the eight-week cycling intervention in schools, control schools will be given the opportunity to conduct the *Bikeability* sessions and will receive all the motivational strategies used for the participating adolescents.

### 2.6 Measurement procedures and outcome measures

#### 2.6.1 Study assessment protocol

**Figure 2:**
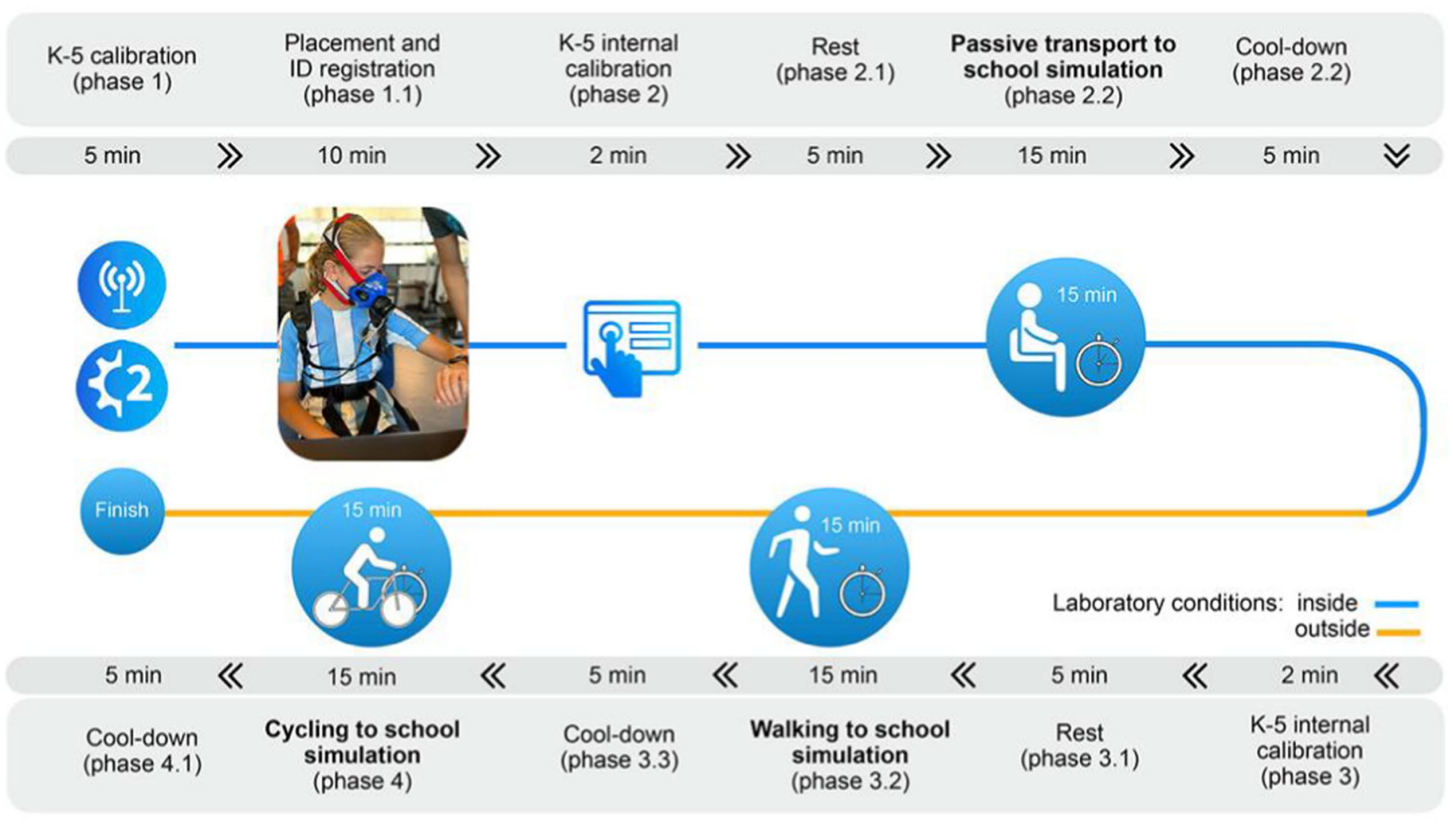
Complete phase I protocol showing the three controlled environment simulations (i.e., commute by motorized-vehicle [from phase 2 to phase 2.2], walking [from phase 3 to phase 3.2], and cycling [from phase 4 to phase 4.1]).

In Phase I, participants will perform three different simulations (i.e., commute by motorized-vehicle, walking, and cycling [see Figure 2]): (1) a simulation of driving to school will be performed by motorized-vehicle. After all the calibration process of the Cosmed K5 (both, flow and gas analyzers will be calibrated before every gas exchange measurement), a face-mask attached to the K5 will be place and gas exchange will be check during a two-minute period before the measurement start. After the check phase, gas exchange recording start during a five-minute period, while participants remain seated. Then, the participants will simulate commute in a motorized-vehicle by remaining seated in a chair for another 15-minute period, having previously walked for 2 mins simulating that participants are moving toward the motorized-vehicle from home. After this first simulation, there will be a five-minute period of cooling down. The total time of this first test will be 27 min. (2) A simulation walking to school will be conducted in a closed circuit defined by the research staff. The same procedures mentioned before will be followed. Then, the participant will walk for 15 min (the participant will determine the speed and intensity of walking) and finish with a five-minute cool down; (3) Participants will simulate a cycling route. They will wait for five-minute after walking and they will start cycling during 15-minute period along the designated route. The simulation will finish with a five-minute cool down period. The total time used in the walking and cycling simulations will be 45 min. These times were chosen because an average walking speed of 4.4–4.6 km/h would not exceed the distance Spanish adolescents are willing to walk to their educational center (1,350 m) (52). While no study in Spanish adolescents indicates the distance, they are willing to cycle to school, we estimated a distance of 4,200 m, the threshold distance used for Australian adolescents (53), with which a speed of approximately 15 km/h would not exceed 15 min. Detailed visual information on the evaluation and calibration process of the Cosmed K5 (Cosmed, Rome, Italy) can be found at the following link.^1^ In order that one active mode of commuting (e.g., walking) does not affect the post exercise oxygen consumption, thus PAEE, of the subsequent active mode of commuting (e.g., cycling), participants will alternate randomly by first performing the simulation of walking or cycling. In addition, the alternation of starting on the bike and walking will be counterbalanced by having the same number of participants starting on the bike or walking. Finally, all data will be downloaded using PC OMNIA.9 software v3.30.79 (Cosmed, Rome, Italy).

**Figure 3:**
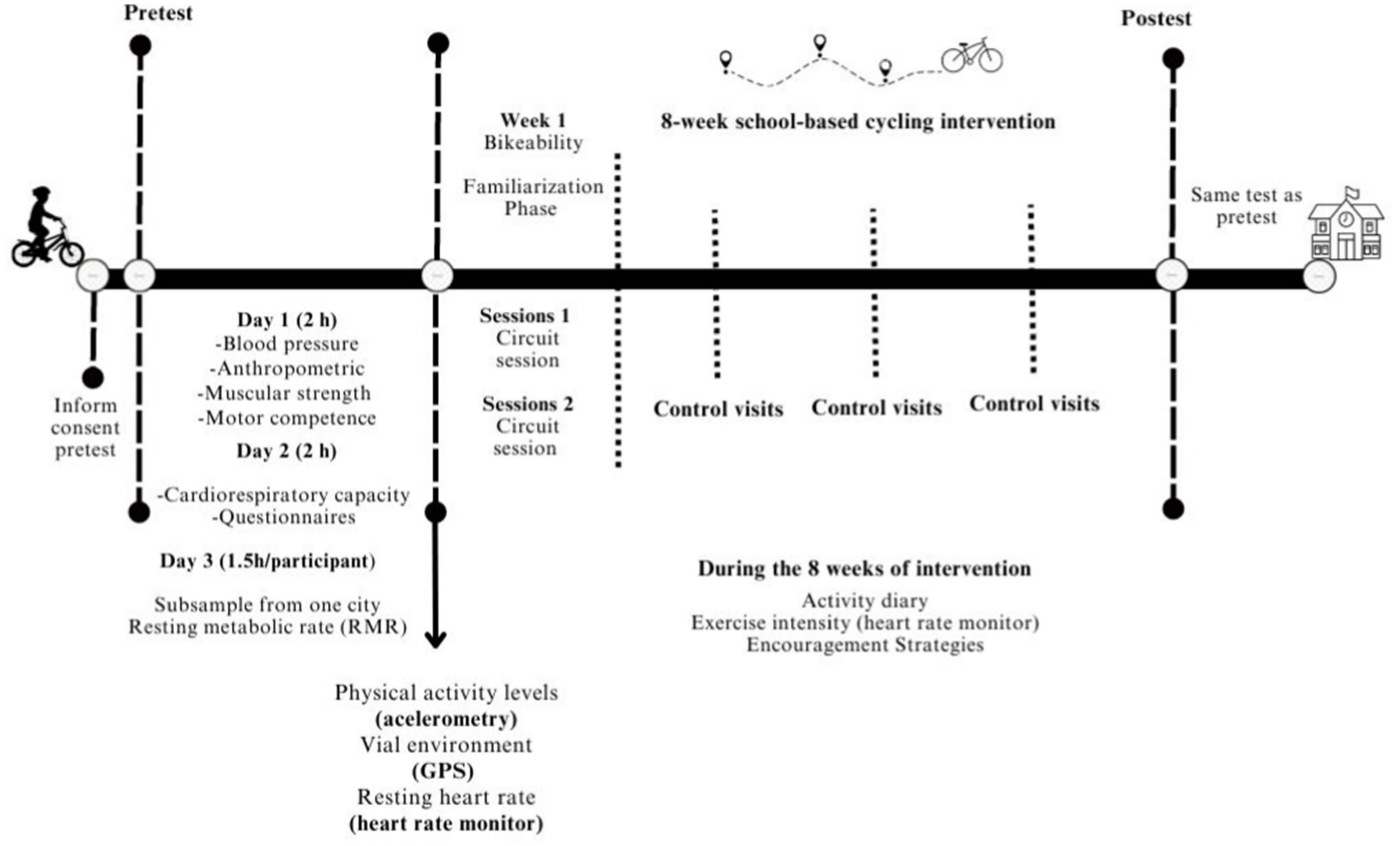
Intervention design phase II of the ENERGYCO study.

In the Phase II, the procedures of the intervention will be tested as a pilot phase to ensure its feasibility and effectiveness. Prior to this, the research team will undergo joint training for internal evaluation. The pilot phase will be implemented in adolescents from a school in Granada over 2 days, with the involvement of the entire research team, lasting approximately 1 h per day. In this pilot phase, the organization of evaluations and the effectiveness of the tests used will be observed by the research group. Moreover, the school-based cycling intervention, which will be described in detail later, will be designed by research personnel trained as Physical Education teachers and graduated in Sport Sciences, and subsequently reviewed by research experts. During the baseline and follow-up of Phase II, the RMR, along with various health-related outcomes, will be assessed. These include individual, social, environmental, and safety factors associated with ACS (see Table 3). Figure 3 outlines the assessments to be conducted during the baseline and follow-up periods.

**Table 3:** Summary of the variables and instruments phase I and PHASE II.

Section	Variables	Instrument	Phase I	Phase II
Primary outcomes				
Physiological outcomes	Resting metabolic rate	Omnical (Maastricht Instruments, Maastricht, The Netherlands)		X
	Energy expenditure	Cosmed K5 (Cosmed, Rome, Italy)	X	X
Secondary outcomes				
Socio-demographic characteristics	*Students:* Age, school grade and class, gender, full postal address, bicycle owners	Student questionnaire	X	X
	*Parents:* School, name, age, gender, children’s gender, and full postal address, parental education level	Family questionnaire		X
Physiological outcomes	Blood Pressure	Stethoscope		X
	Heart rate	Polar Ignite 10, chest band and watch (Polar Electro Oy, Kempele, Finland)	X	X
	Body temperature	Ibuttons (Maxim/Dallas Semiconductor Corp., USA)	X	X
Physical outcomes	*Physical activity levels*	Accelerometer ActigraphGT3x+	X	X
		Youth Activity Profile questionnaire (YAP)		X
	*Physical fitness* Cardiorespiratory capacity			X
	Muscular strength	Course Navette		X
	Physical literacy	Analog dynamometer		X
		Spanish perceived physical literacy instrument for adolescents (PPLI- Q) and Bruininks-Oseretsky Test of Motor Proficiency (BOT-2)	X	X
	*Anthropometric measures* Body mass	Weighting platform (Seca 876, Seca, Ltd., Hamburg, Germany)	X	X
	Height	Height meter (Seca 2013, Seca, Ltd., Hamburg, Germany)	X	X
	Waist and neck circumferences	Non-elastic tape measure	X	X
	*Body composition* Fat free mass, fat mass, lean mass index, fat mass index, body fat percentage, visceral adipose tissue, and bone mineral content	Dual energy X-rayabsorptimetry (DEXA)	X	X
Psychosocial outcomes	Perceived barriers to active commute to school	Barriers for active commuting to school questionnaire (BATACE) and Parental Perception of Barriers toward Active Commuting to School (PABACS)		X
	Autonomy, competence, and relatedness	Basic Psychological Need Satisfaction Need in Active Commuting to and from School questionnaire (BPNS ACS) and Behavioral Regulation in Active Commuting to and from School (BR-ACS)		X
	Satisfaction and motivation in ACS	Positive and Negative Affect Schedule (PANAS) and Rosenberg Self-Esteem Scale (RSES)		X
Other relevant outcomes	*ACS and related outcomes* Usual mode of commuting, frequency of trips to and from school, and independent mobility	PACO questionnaire		X
	Cycling knowledge and cycling skills	Cycling tests		X
	*Family socioeconomic status* Family income	Family Affluence Scale questionnaire (FAS III)		X
	*Parental perceptions* Barriers to allow their adolescent to active commuting to/from school	Parental Perception of Barriers toward active commuting to school questionnaire (PABACS)		X
	Distance home-school	Google Maps and GPS		X
	Trips to and from school characteristics (i.e., distance, speed, and duration)	GPS	X	
	*Weather* Temperature during commuting simulations	iButtons (Maxim/Dallas Semiconductor Corp., USA)	X	
	Temperature (maximum, minimum, mean), total rainfall, and mean wind speed	NationalWeather Data Bank		X
	*School characteristics* Student enrolment, socioeconomic status, and school engagement	Electronic and manual Search, Geographical Information System. Ministry of Education and Vocational Training.		X

ACS, active commuting to and from school; GPS, Global Positioning System.

Assessment day 1 (2): this session will include anthropometry, blood pressure, muscular strength, and motor competence assessment at an indoor sport facility.

Assessment day 2 (2 h): in this session the cardiorespiratory fitness of the participants will be assessed. Once participants have finished the test, they will have to complete an online student-questionnaire. During this session, participants will receive a belt with an accelerometer (GT3X+ accelerometers; ActiGraph, Pensacola, FL) and a Global Positioning System (GPS; QStarz BT-Q1000XT; Travel Recorder, International Co, Ltd. Taipei, Taiwan), a heart rate monitor, including chest band and watch, (Polar Ignite 10, Polar Electro Oy, Kempele, Finland) for measuring their resting heart rate during the first weekend of the baseline week, and to use it during the cycling trips to and from school.

Assessment day 3 (1 h and a half): in this session the RMR of the participants will be assessed. Participants will have to come to the indicated facility in a postabsorptive state (10–12 h without consumption of caloric food or beverages) and by passive transport. Moreover, participants from the experimental group will be provided with two links, one to fill in weekly, after the *Bikeability* sessions week, providing information about their weekly cycling experience to and from school and their opinions on the motivational strategies, and another link with access to a questionnaire to be filled in by their parents.

### 2.7 Outcome measures

#### 2.7.1 Primary outcomes

##### 2.7.1.1 Resting metabolic rate

RER will be measured using the Omnical metabolic cart (Maastricht Instruments, Maastricht, The Netherlands), a validated system for assessing volumes of oxygen consumption (VO_2_) and carbon dioxide production (VCO_2_) during resting conditions which a low measurement error for determining RER (1.7 ± 0.9%) (54). During the test, a ventilated plastic canopy will be connected to the instrument and placed over the participant, allowing the individual to breathe normally while the Omnical records CO_2_ and O_2_ levels in inhaled and exhaled air every 5 s. The device will be calibrated daily using a gas mixture of 18.0% O_2_ and 0.8% CO_2_. Data will be analyzed as the average VO_2_ (ml/min) and VCO_2_ (ml/min) over a 10-min stable period, aggregated by participant at baseline and follow-up. However, since the RMR measurement can only be performed at the research center in one of the participating cities, a contingency plan has been established in case participants from other cities cannot travel to the evaluation center. In such cases, the RMR will also be estimated for all participants using the Schmelze equation, which has demonstrated superior results compared to the gold standard among 12 equations, including a low measurement difference with the gold standard (−51.0 to 15.0 kcal/day), low % bias (−1.6 to 5.8%), an acceptable prediction percentage relative to the gold standard (40.7 to 59.0%), and a low RMSE (136.2 to 305.4 kcal/day) for adolescents with normal weight, overweight, or obesity (55).

##### 2.7.1.2 Energy expenditure related to active commuting to school

PAEE related to different modes of commuting to school (i.e., walking, cycling, passive modes) will be measured using the Cosmed K5 portable metabolic system in Phase I. The Cosmed K5 has previously shown a good level of agreement compared to stationary metabolic carts at low cycling intensity (5.8% difference in VO_2_). At moderate intensities, it slightly underestimated VO_2_, VCO_2_, and energy expenditure (~6.6–6.9%). Additionally, during walking tests, the Cosmed K5 demonstrated high reproducibility, with a coefficient of variation of 4.5% for energy expenditure per kilometer and a concordance coefficient of 0.91. VO_2_ measurements were consistent, with low variability in the fraction of expired oxygen (coefficient of variation of 0.9%) (56). The Cosmed K5 is worn on the back with a harness and measures oxygen consumption (VO_2_) and carbon dioxide production (VCO_2_) in breath-by-breath mode. Before each assessment, the device will be calibrated using a reference gas mixture of 15.92% O_2_ and 5.03% CO_2_. Delay calibration for the breath-by-breath mode will also be performed (57). During the test, participants will breathe normally while commuting, and the Cosmed K5 will record data continuously. Data will be analyzed as the mean VO_2_ and VCO_2_ (ml/min) during each commuting mode. In Phase II, PAEE related to cycling will be estimated using predictive equations developed in Phase I. These equations will incorporate variables such as distance, speed, and heart rate, and will be cross-validated against PAEE measurements obtained with the Cosmed K5 to ensure accuracy. Therefore, data will be analyzed as the mean VO_2_ and VCO_2_ (ml/min) during each cycling trip to and from school, aggregated by participant during the intervention period.

#### 2.7.2 Secondary outcomes

##### 2.7.2.1 Socio-demographic characteristics

Adolescents will self-report their gender, age, school grade, full postal address, socioeconomic status (including family education level and parents’ professions), extracurricular activities, and bicycle ownership through a structured questionnaire. Moreover, their parents will have to complete another questionnaire, where they will self-reported similar information to those previously self-reported by the adolescents. These data will be aggregated by participant and family, and they will be collected during baseline and follow-up assessments.

##### 2.7.2.2 Heart rate

Heart rate will be monitored during three different commuting simulations in Phase I using the Polar Ignite H10 device (Polar Electro Oy, Kempele, Finland), which includes a chest strap and a watch. The Polar Ignite H10 has been considered the gold standard for measuring heart rate in sports conditions (with an RR interval mean bias ± limit of agreement of 0.2 ± 26.8 ms compared to a Holter monitor) (58). Data will be continuously recorded during the simulations and downloaded using the Polar app on a computer. The average heart rate (beats per minute) across the simulations will be calculated and aggregated by participant during baseline assessments. In Phase II, participants will measure their resting heart rate in the morning during the first weekend of both baseline and follow-up. Additionally, they will monitor their heart rate during each cycling trip to and from school throughout the eight-week intervention period. Data will be downloaded by the research staff using the Polar app after the follow-up period. The mean heart rate during cycling trips will be analyzed and aggregated by participant to examine trends over the intervention period.

##### 2.7.2.3 Blood pressure

Blood pressure will be evaluated using a standardized protocol for children (59), that employs the auscultatory method. A stethoscope will be placed on the elbow flexure over the radial artery to determine systolic blood pressure (SBP) and diastolic blood pressure (DBP). Measurements will be conducted individually for each participant and averaged across three repeated assessments at baseline and follow-up.

##### 2.7.2.4 Body temperature

Body temperature will be measured using an iButton device (DS-1922 L, Thermochron; resolution: 0.0625°C; Maxim, Dallas, USA) placed on the left wrist with an epoch of 1 s. During sports conditions, the iButton device observed error estimates were within acceptable limits for the skin temperature method comparison, with typical errors of <0.3°C (showing an r coefficient of >0.999 under all assessed conditions) (60). Data will be analyzed using the Temperatus software^2^ (61) and aggregated as the mean temperature over the recording period for each participant at baseline and follow-up.

##### 2.7.2.5 Physical activity levels

PA will be objectively assessed using an ActiGraph GT3x + accelerometer (ActiLife, version 6.11.7) worn on the hip for seven consecutive days during waking hours, excluding bathing, water activities, or sleeping. The accelerometer records acceleration in three axes, reporting the amount of activity performed at three intensity levels: light, moderate, and vigorous. Data will be processed using the open-source R package GGIR v.3.1–2. The processing pipeline includes: (i) auto-calibration of raw accelerometer data based on local gravitational acceleration (62), (ii) detection and imputation of non-wear time (63), and (iii) calculation of activity counts over 15-s epochs using the algorithm described by Neishabouri et al. (64) and facilitated in the open-source R package actilife counts (DOI: 10.5281/zenodo.7723333), and classifying MVPA as every epoch above the established cut-point for MVPA of 574 counts/15 s (65). Data will be aggregated by participant as the average daily time spent in MVPA during the baseline and follow-up assessments. In addition, PA will be self-reported using the Spanish Youth Activity Profile questionnaire a validated tool for estimating PA and sedentary behaviors at the group level in adolescents (kappa = 0.61–0.77; Intraclass Correlation Coefficient [ICC] = 0.77–0.89) (66). The Spanish version developed using a back-translation process, includes 19 items divided into three sections: (i) activity at school, (ii) activity out-of-school, and (iii) sedentary behaviors. The final variables to be analyzed will include average scores ranging from 1 to 5 for PA at school and PA outside of school, aggregated by participant during baseline and follow-up assessments.

##### 2.7.2.6 Physical fitness

Cardiorespiratory fitness will be assessed using the validated Course Navette field test (67). Adolescents will run between two lines separated by 20 meters, maintaining a rhythm dictated by audio signals from a pre-recorded CD. The initial speed will be 8.5 km/h, increasing by 0.5 km/h per minute. The test will end when participants fail to reach the lines according to the audio cues for at least two consecutive trials or stop due to fatigue. The equations by Léger et al. (67), previously validated in children and adolescents (*r* = 0.587) (68), will be used to estimate maximum oxygen consumption (VO_2_max), calculated individually for each participant during baseline and follow-up assessments. Muscle strength of the upper and lower limbs will be evaluated using standardized and validated protocols (69, 70). Upper limb strength will be measured using an analog dynamometer (Takey TKK5401) following the ALPHA fitness battery recommendations. Participants will perform the test with both hands. Lower limb strength will be assessed using the Standing Long Jump (SLJ) test, a valid and reliable tool for measuring lower limb power (70). Both handgrip strength and SLJ presents a high association with isokinetic parameters (*r* = 0.61 and 0.87; R^2^ = 0.39 and 0.76, respectively) (71). Additionally, the mid-thigh pull test (IMTP) will be conducted, an isometric test of maximum leg and back strength performed with an analog dynamometer (Takei TKK5402). This test has been previously validated by considering different knee and hip positions, showing moderate reliability between positions (r ranging from 0.666 to 0.739) and high reliability between sessions (*r* > 0.819) (72). For all strength tests, measurements will be conducted twice with a two-minute rest between trials. Data will be averaged across trials and aggregated by participant during baseline and follow-up assessments.

##### 2.7.2.7 Physical literacy

Perceived physical literacy will be evaluated using the Perceived Physical Literacy Instrument (PPLI). The PPLI has shown convergent validity ranging from 0.40 to 0.52, indicating that its three factors present adequate discriminant validity and moderate to good reliability (ICC = 0.62–0.79) (73). This tool consisting of nine items designed for adolescents and scored on a five-point Likert scale (1 = strongly disagree to 5 = strongly agree). The nine items are equally divided into three subscales: (a) knowledge and understanding (three items), (b) self-expression and communication with others (three items), and (c) sense of self and self-confidence (three items) (73). The final variable will be the mean score of the nine items aggregated by participant during baseline and follow-up.

##### 2.7.2.8 Motor competence

Motor competence will be assessed using the Bruininks-Oseretsky Test (BOT-2; ICC >0.76 for all items) (74). The short form of the test will be used, consisting of 14 subtests that evaluate fine and gross motor skills. Fine motor tasks include Drawing, Lines through Winding Paths, Folding Paper, Copying a Square, Copying a Star, and Transferring Pennies. Gross motor tasks include Hopping on One Foot, Same-Side Synchronized Hopping, Heel-to-Toe Walking on a Line, Standing on One Leg and Balance Beam (Eyes Open), Stationary One-Legged Jumping, Catching a Ball with Both Hands, Dribbling a Ball, Alternating Hand Movements, Knee Push-Ups, and Sit-Ups. These tasks measure fine motor precision, bilateral coordination, balance, running speed, agility, upper body coordination, and strength (75). Results will be standardized using normative data from the BOT-2 Test Manual and aggregated by participant during baseline and follow-up assessments.

##### 2.7.2.9 Anthropometric measures

Anthropometric measurements will include body mass, height, Body Mass Index (BMI), and circumferences of the waist and neck. Body mass and height will be measured with participants wearing appropriate Physical Education class clothing (shorts and a short-sleeved shirt) and bare feet, using a SECA scale and stadiometer (model 799, Electronic Column Scale, Hamburg, Germany). Body mass will be recorded to the nearest 0.1 kg, and height will be measured in the Frankfort plane to the nearest 0.1 cm. Sitting height, used to estimate biological or somatic age, will also be measured using a stadiometer (Holtain Ltd., Crymmych, Pembs, United Kingdom) equipped with a sit-down drawer. BMI will be calculated as body mass divided by squared height (kg/m^2^) and classified into normal weight, overweight, or obesity categories based on Cole et al.’s cut-off points (76). Waist and neck circumference will be measured using the Executive thin line W606pm tape measure (Lufkin, Mexico). Two consecutive measurements will be taken for each circumference and averaged. All anthropometric measurements will be conducted during baseline and follow-up assessments.

##### 2.7.2.10 Body composition

Body composition will be assessed using Dual-energy X-ray Absorptiometry (DXA; Hologic Series Discovery QDR scanner [Bedford, MA], and APEX software, version 4.0.2). DXA is a scanner with high precision for determining body mass (ICC:0.999), lean mass (0.995), fat mass (0.998), and bone mineral content (0.996) (77). The DXA scan will provide data on fat-free mass, fat mass, lean mass index, fat mass index, body fat percentage, visceral adipose tissue, and bone mineral content. In addition, total body less head values will be reported, following the recommendations of the International Society of Clinical Densitometry for pediatric populations (78). Measurements will be conducted individually for each participant during baseline and follow-up assessments.

##### 2.7.2.11 Perceived barriers to ACS

Perceived barriers to ACS will be assessed among participants using the BATACE Scale (79). Indeed, the BATACE scale has been considered a validated and reliable tool for evaluating barriers to ACS in Spanish adolescents (*α* ranged from 0.64 to 0.72; and ICC between test and retest ranged from 0.68 to 0.77) (79). The scale includes 18 items that address two key dimensions: environmental/safety factors and planning/psychosocial aspects. The resulting variable for analysis will be the average score across all items, ranging from one to four, where one indicates a low perception of barriers and four indicates a high perception of barriers. Scores will be aggregated by participant and collected during baseline and follow-up assessments. Parental perceived barriers to ACS will be evaluated using the PABACS (Parental Perception of Barriers toward ACS) questionnaire, a validated (rho ranged from 0.20 to 0.31) and reliable tool (ICC ranged between test and retest ranged from 0.51 to 0.55) (80). This questionnaire consists of 23 items divided into three scales: general barriers, walking barriers, and cycling barriers. The final variables for analysis will be the average scores for each scale and individual items, ranging from one (strongly disagree) to four (strongly agree). Data will be aggregated by parent during baseline and follow-up assessments.

##### 2.7.2.12 Autonomy, competence, and relatedness related to ACS

Basic psychological needs satisfaction in ACS will be assessed using Basic Psychological Need Satisfaction in ACS Scale (BPNS-ACS) (81). The BPNS-ACS scale have shown a good internal consistency (*α* ranged from 0.81 to 0.92) and a high ICC between test and retest (ICC ranged from 0.76 to 0.81) (81). This scale consists of 12 items that explore participants’ thoughts and feelings about their usual mode of commuting to and from school. Participants will rate their agreement with each item on a five-point Likert scale, ranging from “strongly disagree” to “strongly agree.” The resulting variable will be the average score across all 12 items, aggregated by participant and collected during baseline and follow-up assessments. Motivation for ACS will be evaluated using the Behavioral Regulation in ACS (BR-ACS) questionnaire (82). This scale presents both a high internal consistency (α ranged from 0.70 to 0.91) and ICC (ranged from 0.70 to 0.76) (83). The BR-ACS consists of six factors and 23 items that assess different forms of motivation, including intrinsic motivation, integrated regulation, identified regulation, introjected regulation, external regulation, and amotivation. Participants will indicate their agreement with statements such as “I go or would go to and from school walking or cycling because.” on a five-point Likert-type scale, ranging from “not true for me” to “very true for me.” The final variables for analysis will include average scores for each of the six factors, aggregated by participant and measured during baseline and follow-up assessments.

##### 2.7.2.13 Self-esteem and mental health

Self-esteem will be assessed using the validated Spanish version of the Rosenberg Self-Esteem Scale (RSES), consisting of 10 items that evaluate self-respect and self-acceptance (84). This tool has demonstrated satisfactory psychometric properties, with Cronbach’s alphas ranged from 0.83 to 0.86, as well as factorial equivalence was validated using a structural equation model (CFI = 0.912; RMSEA = 0.079), indicating a high degree of invariance (84). In this case, participants will rate their agreement with each item on a four-point Likert scale ranging from one (“strongly disagree”) to four (“strongly agree”). Items 1, 3, 4, 7, and 10 measure positive self-esteem, while items 2, 5, 6, 8, and 9 measure negative self-esteem. The resulting variable will be the average score across all items, with higher scores indicating higher self-esteem. Data will be aggregated by participant and collected during baseline and follow-up assessments. Mental health will be evaluated using the PANASN questionnaire (85), a self-report tool with 20 items which presented a good internal consistency (*α* ranged from 0.75 to 0.76) and an acceptable temporal stability (ICC = 0.84). Ten items assess positive affect (e.g., “I am a cheerful person, I tend to get excited”), and 10 items assess negative affect (e.g., “I feel nervous”). Participants will complete the questionnaire based on how they usually feel and/or behave, using a three-point response scale: “Never” (i), “Sometimes” (ii), and “Often” (iii). The final variables for analysis will be the average scores for positive and negative affect, aggregated by participant and measured during baseline and follow-up assessments.

##### 2.7.2.14 Mode of active commuting to school and related outcomes

ACS will be evaluated using the PACO questionnaire^3^, a reliable and valid tool for young Spaniards (Kappa ranged from 0.42 to 0.94) (86, 87). The PACO questionnaire consists of four self-report items designed to assess the usual mode of commuting and the weekly frequency of commuting among children and adolescents. The four questions include: (i) *“How do you usually get to school?”* (ii) *“How do you usually get home from school?”* (iii) *“How did you get to school each day?”* and (iv) *“How did you get home from school each day?”* Participants will select their mode of commuting from predefined options such as walking, cycling, car, motorcycle, bus, public bus, metro/train/tram, or other (requiring mode specification). The main variables of interest are the percentage of usual cycling and active modes of commuting to and from school, along with the weekly number of cycling and active trips. Additionally, participants will report their distance from school and the time it takes to commute from home to school. Response options for distance range from less than 0.5 km to 5 km or more, while response options for travel time range from less than 15 min to 60 min or more (86). Other factors associated with ACS, such as independent mobility, will also be analyzed as secondary variables. Data will be aggregated by participant and collected during baseline and follow-up assessments. Overall, these questionnaires provide valuable data on modes of commuting, frequency of active commuting, distance covered, and time spent during the commute for both adolescents.

##### 2.7.2.15 Cycling skills

Cycling skills will be assessed using a specific observational checklist completed by the research staff after each participant’s performance during the *Bikeability* session. The checklist includes 18 cycling skills grouped into six categories: (i) starting off, pedaling, and changing gears; (ii) turning right; (iii) turning left; (iv) performing zigzags; (v) braking; and (vi) unexpected turning. Each skill is scored dichotomously (yes/no), and participants will receive a final score ranging from 0 to 18 points. Data will be aggregated by participant and collected during the *Bikeability* session. The checklist has been previously published, and it is available elsewhere (88).

##### 2.7.2.16 Family socioeconomic status

Family socioeconomic status will be assessed using two complementary tools. Adolescents will self-report their family’s socioeconomic status through the Family Affluence Scale III (FAS III; *r* = 0.90 and ICC >0.76) (89). The resulting variables will include composite scores derived from the respective scales, reflecting the level of family affluence. Data will be aggregated by participants and family, and collected during baseline and follow-up assessments.

##### 2.7.2.17 Home-school distance

Distance from home to school will be determined using two objective methods. The first method calculates the shortest walking path between the participant’s home and school using Google Maps™ software (52). The second method combines GPS and Geographical Information System (GIS) data to establish the actual route taken by participants between home and school. Additionally, participants and parents will self-report their postal addresses, and the school’s postal address will also be obtained. These addresses will serve as reference points for both methods. The resulting variables will include the calculated shortest distance (km) and the actual route distance (km). Data will be aggregated by participant and collected during baseline and follow-up assessments.

##### 2.7.2.18 Trip characteristics

ACS behavior will be objectively evaluated over seven consecutive days using a Q-STARZ BT-Q1000XT GPS device, manufactured by Int Co., Ltd. (Taipei, Taiwan). The GPS will record distance, speed, and trip duration for each commute performed by the participants. Additionally, environmental variables such as terrain slope will be measured and later used as confounders in the analysis. The GPS will be configured with a 15-s epoch to ensure precise data collection. This protocol, which combines GPS data with accelerometry for analyzing ACS behavior, has been previously validated by our research group (17, 20, 31). In this project, the combination will also be used to estimate PAEE associated with different modes of commuting to school. The main variables of interest include distance (km), average speed (km/h), and trip duration (minutes). Data will be aggregated by participant and collected during baseline and follow-up assessments.

##### 2.7.2.19 Weather

Ambient and weather conditions will be assessed using two complementary methods. In Phase I, ambient temperature will be measured using iButtons (S-1922 L, Thermochron; resolution: 0.0625°C; Maxim, Dallas, USA). Participants will wear an iButton attached to their trousers to measure outdoor temperature. It is important to note that the iButton device has also demonstrated a very high correlation with a mercury thermometer (r > 0.999) for measuring ambient temperature (REF). Data from the iButtons will be analyzed using the Temperatus software (see Footnote 2) (90). In Phase II, temperature (maximum, minimum, and average), total rainfall, and average wind speed will be obtained from the National Weather Data Bank. This open-source database contains climatological information recorded by observatories across the country. Data will be collected from the weather station closest to each school for the five weekdays and the preceding weekend prior to completing the student questionnaire. These variables will be recorded at both baseline and follow-up. The main variables of interest include daily ambient temperature (°C), total rainfall (mm), and average wind speed (km/h). Data will be aggregated by participant and city.

##### 2.7.2.20 School characteristics

School characteristics will be assessed using various measures. School size will be determined based on student enrollment, reflecting the total number of students enrolled during the study period. School socioeconomic status (SES) will be established using data from previous studies (91). Additionally, the socioeconomic status of the school neighborhoods will be objectively analyzed and classified as high or low using GIS. Moreover, the length of the cycling lane network within a 5.1 km street-network buffer around each school will be calculated using GIS (92). Additionally, school staff will be asked to provide information on the availability of bicycle racks either on school premises or near the main entrance. School engagement, which may influence the effects of the intervention, will be assessed through the perceptions of the research staff. Engagement will be categorized into three dimensions: teacher engagement (average staff perceptions of teacher involvement across all classes), student engagement (average staff perceptions of student participation across all classes), and overall school engagement (average of teacher and student engagement scores). Data for all variables will be collected during baseline and follow-up assessments and aggregated by participant and school.

### 2.8 School-based cycling intervention program

The school cycling intervention program will include 1 week of training and familiarization with the bicycle using the *Bikeability* methodology (88), and the adolescents’ commuting to and from the school for 8 weeks with the support of encouragement strategies during the intervention. *Bikeability* sessions will have the objective of familiarizing the students with cycling to and from school. This is based on the fact that participants will know how to cycle, but many of them might not have cycled for a long time and have not cycled regularly. Moreover, *Bikeability* sessions will help the research staff to analyze if any student does not have sufficient capacity to carry out the intervention. The *Bikeability* methodology, previously tested and published as innovative material by our research group (88), will consist of two sessions over a one-week period delivered by the research group with the support of the Physical Education teacher during Physical Education class to acquire practical skills and understand how to cycle safely and effectively. The components and content of these two sessions focus on: (i) knowledge about road safety regulations, safety equipment for cyclists, hand signals while cycling, and the benefits of cycling as a means of transportation, and (ii) cycling-related skills. Prior to the first session, the Physical Education teacher will provide students with educational theoretical material to raise awareness about the benefits and usefulness of cycling as a mode of travel, knowledge about road safety rules for cyclists, knowledge about safety equipment for cyclists for both the cyclist and the bicycle, and hand signals while cycling in an urban context. The sessions conducted by the research group with the support of the Physical Education teacher are described below:

1. Closed Circuit Session (60 min—first day): A practical session will be conducted at the school facilities to learn or improve the fundamental cycling skills necessary for safe biking. Participants will practice how to properly use a helmet and inspect the bicycle before riding, as well as fundamental cycling skills through 10 activities and a cycling circuit. The content will include proper helmet fitting, bicycle safety check before riding, fundamental cycling skills for starting and pedaling, braking safely, shifting gears, and hand signals for changing direction.
2. Closed Circuit Session (60 min—second day): During the second session, the knowledge about the use and maintenance of bicycles will be reviewed, in addition to the cycling skills practiced in session one for their corresponding evaluation. It is important to note that the cycle skills results will be highly dependent on participants’ prior cycling skills, but this session will help to reinforce the skills of those who do not cycle regularly.

The participating schools will provide the necessary materials for the *Bikeability* sessions, such as signaling objects (cones and hoops) to build the circuits. Regarding the methodology, the sessions will encourage student participation, and the language used for communication will be inclusive and adapted to them. Active participation of the students will be promoted through questions, fostering dialog, and providing meaningful individual and group feedback. Additionally, based on previous interventions (45), some alternative strategies will be considered for implementing the *Bikeability* methodology: (i) half of the students will perform the activities first, followed by the other half; (ii) students from one class can lend their bicycles to students from another class; (iii) some secondary schools have their own bicycles that are offered free of charge to students; and (iv) local transport organizations or transportation can be asked to lend bicycles for the intervention. Detailed visual information on the *Bikeability* sessions can be found at the following link.^4^

The second part of the intervention will consist of an eight-week program cycling to and from school. The duration of 8 weeks for school cycling is appropriate in this setting according to a previous systematic review (47). During the eight-week intervention, adolescents will receive an encouragement strategy every week in the form of a challenge related to knowledge content about the importance of PA and having good physical condition for our health, benefits of ACS, knowledge of RMR, PAEE, and its importance, promotion of confidence and motivation for adolescents to travel safely in their environment. The functioning of these encouragement strategies, called Octopus Strategy, will consist of eight games that will be opened weekly as the intervention progresses. In each of the games a series of points will be obtained, and these will be accumulated so that there will be a competition between the different participating schools. The winner of the Octopus strategy will be the participant who completes all the games during the 8 weeks. It is important to highlight that although motivational strategies will be employed to support adherence to the intervention, they are not intended to address broader behavior change processes, such as altering participants’ attitudes or intentions toward active commuting. Thus, the Octopus strategy will aim to improve the adherence and motivation to the intervention (i.e., to cycle at least 3 days to and from the school). Table 4 shows a summary of the project’s objectives related to the *Bikeability* sessions and the contents to be developed in the encouragement strategies, during the eight-week intervention. More information about the different challenges proposed by our research group as an encouragement strategy in the Supplementary material and in the following link.^5^

**Table 4:** Eight-week school-based cycling intervention overview: octopus strategy.

Activity		Aim	Content	Instrument (target)
1 (week 1 and 2)	Training 1:Bikeability	2, 4 & 5	Implementation of the basic guidelines for bicycle maintenance, in addition to the rules for entry, exit and parking of the bicycle Knowledge and practice of cycling skills on a circuit closed to traffic Practice driving skills on closed circuits to traffic	Bicycles, helmets, first aid kits, cones, and whistle. Student skills evaluation sheet (adolescents/teachers)
2 (week 2)	Encouragement strategies	2	Importance and knowledge of energy expenditure	Infographics (adolescents/teachers)
3 (week 3)	Encouragement strategies	2	Knowledge of World Health Organization recommendations on daily physical activity, as well as its benefits Importance of good physical condition for health	Infographics (parents/adolescents/school center/teachers)
4 (week 4)	Encouragement strategies	1 & 2	Contribution of active commuting to/from school on daily physical activity, as well as its benefits	Infographics (parents/adolescents/school center/teachers)
5 (week 5 and 6)	Encouragement strategies	3, 4 & 5	Positive messages and advice on safe routes for cycling (determining factors of intention or attitude)	Motivational videos (parents/adolescents/school center/teachers)
6 (week 7)	Encouragement strategies	4 & 5	Traveling with family and friends in a fun and safe way	Motivational videos (parents/adolescents)
7 (week 8)	Encouragement strategies	3, 4 & 5	Confidence and motivation to cycling (behavioral control)	Motivational videos (parents/adolescents/school center/teachers)

### 2.9 Adverse events

Cycling is generally recognized as a safe and well-tolerated form of PA for adolescents. However, any potential adverse events will be carefully monitored throughout the intervention period. Minor adverse events not directly related to the intervention (e.g., seasonal allergies, weather) and those commonly associated with PA (e.g., fatigue) will not be systematically recorded unless they lead to a modification of the intervention protocol (e.g., the participant is unable to cycle to and from school at least 3 days/week). In such cases, the principal investigator will review the situation and decide whether the participant can continue in the study. On the other hand, in the event of any significant health-related issues, such as a serious injury, these incidents will be assessed by the research team. Again, the principal investigator will review the case and make informed decisions regarding participant safety, including whether the participant can remain in the study. If the participant manages to recover in time and is able to cycle to and from school 3 days/week, he/she will not be allowed to return to the intervention without providing medical clearance to support their resumption of PA. The safety and well-being of participants remain the primary focus of the research team. All adverse events will be systematically monitored, recorded, and reported in future manuscripts to ensure transparency and accountability. To achieve this, the research team will maintain a dedicated document to capture details such as the type, duration, and resolution of adverse events.

### 2.10 Data analysis

#### 2.10.1 Analysis populations

In the ENERGYCO project, per-protocol and intention-to-treat (ITT) approaches, as well as sensitivity analyses, will be conducted. On one hand, the per-protocol approach will be used to present the main and secondary analysis. Participants from the intervention group who have cycled to and/or from school at least 3 days/week over 8 weeks will be included. Additionally, participants from both the intervention and control groups must have valid data at baseline and follow-up for the outcomes of interest (e.g., RMR and PAEE). On the other hand, the ITT approach will be used to present main and secondary analysis, but in the Supplementary material. This approach will include all participants in both the intervention and control groups who have valid data at baseline and follow-up or who have missing data at either baseline or follow-up. Finally, the potential impact of missing data will be assessed using various imputation models and sensitivity analyses. Specifically, we will conduct sensitivity analyses to determine whether the choice of imputation method affects the results. Additionally, no interim analyses will be performed.

#### 2.10.2 Statistical analysis

Descriptive analyses of all study variables presented with the mean (standard deviation) and frequencies (percentage) will be performed. Subsequently, the normality of the variables will be analyzed using histograms and normality tests. In Phase I, for the comparison of PAEE between the different modes of commuting (i.e., walking, cycling, and motorized-vehicle), a repeated measures ANOVA will be performed using the per-protocol approach. In addition, Student t-test or Mann–Whitney tests will be performed to compare the fluctuation of PAEE according to speed or distance commuted between active modes of commuting (i.e., walking vs. cycling).Within Phase II, the effects of the school-based cycling intervention on RMR and other health-related outcomes will be studied using an analysis of covariance (ANCOVA). Post-intervention RMR and other-health related values will be included as dependent variables, group (i.e., experimental group vs. control group) as fixed factor, and baseline outcome data as covariates. To evaluate the effect of the intervention on the secondary outcomes (i.e., physiological, physical, and psychosocial outcomes), we will conduct the same analysis used for the main outcomes (i.e., ANCOVA analysis using the intention-to-treat approach). Moreover, to control the variability between schools, the analyses will include adjustments for contextual and organizational variables, such as the SES of the school area, the length of cycling lanes, the availability of bicycle parking, and the level of engagement of teachers and students in the intervention. The estimates will be present as raw and standardized (z-scores; i.e., mean differences between follow-up and baseline data divided by the standard deviation), as an indication of the change the post-intervention z-scores will be calculated relative to the baseline mean and standard deviation. Effect sizes will be categorized as small (0.2 SDs), medium (0.5 SDs), and large (0.8 SDs), based on Cohen (93). Finally, to analyze the influence of covariates on the association between the intervention and the main outcomes (i.e., RMR and PAEE), mediation analyses will be performed. The intervention will be included as the independent variable, and the main outcomes as the dependent variables. Covariates included in the mediation analysis will encompass body composition, physical function, cognitive determinants, perceived barriers or autonomy, built-environment characteristics, weather, among others. In the statistical analysis, a value of *p* < 0.05 will be considered statistically significant.

### 2.11 Data management

The ENERGYCO project will utilize three main systems to store and manage data. First, the Dropbox platform will be used to manage all project-related information, including monitoring documents, evaluation protocols, participant tracking sheets, adverse event control sheets, and raw data for all analyzed variables (both primary and secondary), organized into separate spreadsheets based on the variable being studied. Second, all data will be stored on an external hard drive, kept securely in the principal investigator’s office. The same data and folders stored in Dropbox will also be mirrored on this physical hard drive. Third, informed consent forms, paper-based questionnaires, and participant evaluation sheets will be collected and stored in paper format in the principal investigator’s office. To ensure participant anonymity, each individual will be assigned a unique code upon submitting their informed consent, which will be used in all data reports. The principal investigator will have continuous access to the final dataset and the research staff undertakes not to store on its computer any data related to the ENERGYCO project. Regarding data quality control, the process will include double-checking paper-based evaluation sheets and reviewing the ranges and valid values in the resulting spreadsheets. The research team will perform quality controls and archive the data following a pre-established protocol for both the baseline and follow-up phases. If any data quality issues are detected, the raw data stored in Dropbox or the hard drive will be consulted, along with the paper-based records, which will be reviewed as needed. It is important to highlight that the ENERGYCO project is committed to publishing its findings, whether positive, negative, or inconclusive, in peer-reviewed journals, at national and international conferences, and through public dissemination in local, regional, and national media outlets, as well as on social media platforms. Additionally, these results will be shared with participants and schools through individual reports for participants and comprehensive reports for the schools, including corresponding interpretations of the findings. Finally, eligibility for authorship will be determined by the principal investigator in accordance with the guidelines of the International Committee of Medical Journal Editors (ICMJE), which require substantial contributions to the conception, design, data acquisition, analysis, or interpretation, as well as drafting or critically revising the manuscript and approving the final version.

## 3 Discussion

To the best of our knowledge, the ENERGYCO study will be the first study which will assess the PAEE related to different modes of commuting to school using indirect calorimetry. Moreover, the effects of an eight-week school-based cycling intervention on RMR and PAEE, as well as on other physiological (i.e., basal heart rate, blood pressure, and body temperature), physical (i.e., PA levels, anthropometric measures, body composition, physical fitness, and motor competence), and psychosocial outcomes(e.g., perceived barriers to active commute to school, self-esteem, and mental health) among Spanish adolescents aged will be assessed. The main objective of the study has potential individual implications for adolescents, as the school-based cycling intervention aims to explore its effects on RMR and daily PAEE, which may contribute to changes in PA levels and associated physical, psychosocial, and emotional aspects. However, individual variability and adherence challenges must be considered when interpreting the results. To support engagement, the study will provide easy-to-use encouragement strategies, such as infographics and motivational videos, to promote adherence to the intervention (i.e., cycling at least 3 days to and from school). While these materials may help sustain participation, their effectiveness in maintaining long-term engagement remains to be explored.

The findings of the ENERGYCO study will contribute to the current understanding of the potential effects of a school-based cycling intervention on RMR and daily PAEE among adolescents. Unlike most studies assessing ACS, PAEE, or PA levels, which often rely on self-reported tools, this study will use objective measurement techniques. Nevertheless, current research is incorporating objective devices such as GPS and accelerometry for more precise measurements (94). In this study, in addition to using these devices, indirect calorimetry technique will be employed, adding further value to the research. Furthermore, we will analyze the effects of a school-based cycling intervention on increasing RMR in adolescents, instead of focusing on a behavioral change. Indeed, most interventions in the school context have been focused on changing the behavior of adolescents (36–39), but even though such interventions are necessary, we still do not know in depth the effects on some health variables, especially on physiological outcomes such as RMR and PAEE. Therefore, the ENERGYCO study is expected to provide novel insights into the potential effects of a school-based cycling intervention on RMR and PAEE, along with other physiological, physical, and psychosocial outcomes. As research in this area remains limited, this study will contribute valuable data. Nevertheless, the findings should be interpreted in the context of the study’s duration, sample characteristics, and variability in adherence among participants. In this sense, and following the previous literature, interventions with a duration of 8 weeks could be enough to improve health markers in young people (95, 96).

Furthermore, studies focus on assessing the PAEE related to ACS in free living conditions have used accelerometer counts (20, 97) or heart rate (12) instead of indirect calorimetry. Indeed, as far as we know, two studies focus on young population used indirect calorimetry to measure the PAEE associated to different routine activities (98) or just walking in a treadmill (99), but both of them were carried out under laboratory conditions. The study by Schuna et al. (98) involved 106 American children and adolescents (6 to 18 years old) who completed a series of activities such as sitting, watching movies, coloring, climbing stairs, playing basketball, or jumping. Authors concluded that the lowest average PAEE values were associated with sitting and watching movies, while jumping was the activity that caused the highest average PAEE. While the study by Tudor-Locke et al. (99) evaluated the cadence produced by different walking speeds (an indirect measure of intensity) in young people aged 6 to 20 in a controlled trial. Participants completed multiple 5-min series on a treadmill, ranging from 0.80 km/h to 8.04 km/h. Steps were counted visually, and intensity was objectively measured using a portable metabolic system. Therefore, the ENERGYCO study will contribute to advancing knowledge on PAEE associated with different modes of commuting to school. To our knowledge, this will be the first study to objectively measure PAEE in adolescent commuters using indirect calorimetry in free-living conditions.

The ENERGYCO study aims to provide valuable insights for policymakers, educators, urban planners, and public health professionals by offering concrete recommendations for adapting and implementing similar interventions in different educational and urban settings. In the school context, integrating ACS into curricula through road safety education and awareness campaigns on the health benefits of cycling could enhance engagement. Policymakers should support these efforts by incorporating ACS modules into Physical Education programs and fostering collaborations between schools and local authorities to ensure safe commuting environments. From an urban planning perspective, expanding cycling infrastructure around schools, such as protected bike lanes, traffic-calming measures, and secure bicycle parking, should be prioritized, along with establishing urban design guidelines to improve the accessibility and safety of school routes. Public health initiatives should encourage community-based interventions, including bike-to-school programs, incentive schemes for cycling equipment, and partnerships with local businesses to create cycling-friendly environments. Regarding scalability and sustainability, after the completion of the ENERGYCO study, a comprehensive review of the collected data will be conducted to explore how a more cost-effective assessment approach could be developed. Given the logistical challenges of school settings, this analysis will focus on identifying ways to streamline data collection while maintaining methodological rigor, ensuring feasibility for future studies. The goal will be to refine the assessment of RMR, PAEE, and secondary outcomes in a real-world school environment. To maximize impact, the ENERGYCO study will disseminate its findings through infographics and policy briefs tailored to families, schools, municipalities, and public health entities, ensuring accessibility and usability for decision-makers.

It is important to mention that the ENERGYCO protocol presents some limitations and risks that may arise in the future. These limitations will be: (i) some participants may not attend the assessments, which could lead to a small sample size; (ii) the relatively small sample size in Phase I, which could potentially limit statistical power and generalizability. The sample size was determined based on previous studies using indirect calorimetry in similar research contexts. However, the complexity of the data collection process posed significant challenges for recruitment; (iii) important outcomes such as distance or barriers to cycling to school may also influence the recruitment process, resulting in a low-rate recruitment; (iv) there may be problems with the selection of schools. In Phase II, after the selection of schools, it is possible that there may not be enough students to form each of the groups to low prevalence of cycling to and from school in Spain (46); (v) the family is not involved in the intervention, uncertainties surrounding the help that the school can give to the research group for the success of the intervention (e.g., contact with adolescents). On the other hand, the ENERGYCO protocol presents several strengths. The study design is a cluster randomized controlled trial, as well as the duration of 8 weeks of the school-based cycling intervention, including strategies for maintaining adherence and motivation, and an incentive system. The use of instruments gold standard, such as the Omnical for assessing the RER, or the use of indirect calorimetry for measuring the PAEE related to different modes of commuting will provide accurate data for these variables.

## 4 Conclusion

The ENERGYCO study is designed to advance the understanding of cycling to school and its potential impact on physiological variables through an innovative two-phase structure. Phase I aims to quantify the energy demands of different commuting modes using out-of-laboratory gas exchange instruments, providing objective data on the physiological cost of cycling to school. The results from Phase I may offer valuable insights for schools, families, and practitioners, informing strategies to address sedentary behaviors and promote sustainable ACS, such as walking and cycling to school. Phase II will implement an eight-week school-based cycling intervention targeting Spanish adolescents. This phase will assess its potential impact on a range of outcomes, including physiological, physical, psychosocial, and emotional dimensions. By providing objective data on the short-term physiological effects of cycling to school, the ENERGYCO study aims to contribute to the evidence base supporting ACS strategies for adolescents. These results may help inform effective policies and practices, offering guidance to teachers, families, policymakers, and the clinical sector in implementing scalable and sustainable ACS-focused interventions. Finally, the ENERGYCO study aspires to contribute meaningfully to adolescent health and well-being, encouraging healthier and more active communities.

## Footnotes

^1^ https://www.youtube.com/watch?v=uI6nhUUB19Q

^2^ http://profith.ugr.es/temperatus?lang=en

^3^ http://profith.ugr.es/recursos-paco

^4^ https://drive.google.com/file/d/13vT0QELbFa_itJETI4hHWaBE4QI-f7-T/view?usp=drive_link

^5^ https://view.genial.ly/65a91b8a54833300148e0790/guide-mision-octopus
